# Efficacy and safety of neoadjuvant chemotherapy with immunotherapy versus chemotherapy alone in esophageal squamous cell carcinoma: a meta-analysis based on randomized controlled trials

**DOI:** 10.3389/fimmu.2026.1825905

**Published:** 2026-07-09

**Authors:** Yibang Ye, Liangyu Zhang, Zhenyuan Yang, Yizhou Huang, Maohui Chen, Shuliang Zhang, Taidui Zeng, Chun Chen, Bin Zheng

**Affiliations:** 1 Department of Thoracic Surgery, Fujian Medical University Union Hospital, Fuzhou, China; 2 Key Laboratory of Cardio-Thoracic Surgery (Fujian Medical University), Fujian Province University, Fuzhou, China; 3 National Key Clinical Specialty of Thoracic Surgery, Fuzhou, China; 4 Clinical Research Center for Thoracic Tumors of Fujian Province, Fuzhou, China

**Keywords:** esophageal squamous cell carcinoma, immune checkpoint inhibitors, meta-analysis, neoadjuvant chemoimmunotherapy, neoadjuvant chemotherapy, randomized controlled trials

## Abstract

**Background:**

Esophageal squamous cell carcinoma (ESCC) remains one of the most aggressive and lethal cancers, with high incidence and mortality rates in East Asia. Neoadjuvant chemotherapy (NC) has traditionally been the standard approach for improving resectability in ESCC, but its limited efficacy in achieving complete pathological responses and enhancing survival has driven interest in combining it with immune checkpoint inhibitors (ICIs), resulting in neoadjuvant chemoimmunotherapy (NIC). Based on randomized controlled trials (RCTs), this meta-analysis compares the risks and clinical benefits of NIC versus NC in resectable ESCC patients.

**Methods:**

This meta-analysis systematically reviewed data from randomized controlled trials (RCTs) comparing NIC and NC in the treatment of resectable ESCC. Primary outcomes included pathological complete response (pCR) and major pathological response (MPR), while secondary outcomes focused on overall survival (OS), event-free survival(EFS), surgery rate, microscopically margin-negative resection(R0 resection), Intraoperative and hospitalization indicators, T Staging, Response evaluation criteria in solid tumors(RECIST), and adverse events (AEs).

**Results:**

Six high-quality RCTs (1070 patients) were included. NIC significantly improved major pathological response(MPR) (42.8% vs. 22.2%, Odds Ratio(OR) = 2.40, p = 0.0007) and pathological complete response(pCR) (22.2% vs. 8.0%, OR = 3.53, p < 0.00001). NIC was also associated with borderline statistically significant improvements in overall survival (OS) (HR = 0.57, p = 0.05) and R0 resection rate (OR = 2.56, p = 0.05). In addition, NIC enhanced surgical rate (OR = 1.57, p = 0.02), lymph node resection (Mean Difference (MD) = 1.76, P = 0.008), and NIC with a longer interval to surgery (MD = 4.73, p = 0.003, I2 = 95%), although this pooled estimate is less reliable because of considerable heterogeneity. However, immune-related adverse events (iRAEs) were higher in NIC (23.21% vs. 1.16%, p < 0.00001), though severe AEs were similar.

**Conclusions:**

NIC significantly improves pathological response and other perioperative benefits (e.g., surgical rate and lymph node resection) in resectable ESCC compared with NC, with a trend toward improved survival, despite a higher incidence of irAEs. Further studies are needed to optimize treatment protocols and clarify the long-term impact of immune-related toxicities.

## Introduction

Esophageal squamous cell carcinoma(ESCC) remains one of the most aggressive malignancies worldwide (1), with the highest incidence and mortality rates observed particularly in East Asia (2).According to global epidemiological data, esophageal cancer is a leading cause of cancer-related deaths, with esophageal squamous cell carcinoma (ESCC) being the most prevalent histological subtype (3). Despite progress in early detection, surgical techniques, and perioperative management, the long-term prognosis for patients with locally advanced, resectable ESCC remains poor. This is primarily due to the rapid progression of the disease, high recurrence rates, and limited responses to traditional therapeutic approaches (4, 5).

Neoadjuvant chemotherapy (NC) has been used as one of the perioperative treatment strategies for resectable ESCC, particularly in some Asian clinical settings, with the aim of improving tumor downstaging and facilitating curative resection (6). The goal of NC is to reduce tumor size and minimize the risk of metastasis, thereby enhancing the rate of surgical resection (7). However, the pathological response and long-term survival benefits achieved with neoadjuvant chemotherapy alone remain suboptimal in some patients, which has prompted interest in incorporating immune checkpoint inhibitors into neoadjuvant treatment strategies (8, 9). Some patients fail to achieve satisfactory tumor shrinkage during treatment, with some experiencing early disease progression. Updated comprehensive synthesis of RCT data is needed to clarify the comparative efficacy and safety of neoadjuvant chemoimmunotherapy versus chemotherapy alone in resectable ESCC.

Recent advancements in immune checkpoint inhibitors (ICIs), particularly those targeting the PD-1/PD-L1 pathway, have revolutionized the treatment of ESCC. ICIs restore immune surveillance and have shown remarkable clinical efficacy in various advanced and metastatic cancers (10). The combination of ICIs with chemotherapy in the neoadjuvant setting, known as neoadjuvant chemoimmunotherapy (NIC), is considered a biologically rational and promising therapeutic strategy. By inducing immunogenic cell death through chemotherapy and enhancing immune responses via ICIs, this combination may offer deeper and more durable antitumor immune reactions (11).

Accumulating clinical evidence posits that NIC may confer superior intermediate-to-long-term therapeutic efficacy compared to NC alone. Beyond conventional pathological assessments, the integration of surrogate endpoints, including major pathological response (MPR), pathological complete response (pCR), and radiological metrics such as objective response rate (ORR), has become instrumental in capturing real-time tumor dynamics and early treatment sensitivity (12, 13). These multidimensional markers provide a robust framework for optimizing perioperative strategies and predicting long-term oncological outcomes (14).

Crucially, overall survival (OS) remains the gold standard for evaluating prognostic superiority. Emerging data suggest that while the survival curves of NIC and NC cohorts may converge in the immediate postoperative phase (e.g., at 3 months), a distinct survival advantage favoring the NIC group typically manifests after the 6-month threshold (8). This temporal divergence underscores the potential of immune-checkpoint inhibitors to induce durable anti-tumor surveillance and sustained clinical benefits (15).

Furthermore, the clinical utility of NIC is inextricably linked to perioperative variables, including R0 resection rates, lymph node resection and T-stage downstaging (16). Although NIC has demonstrated a potent capacity for local tumor debulking and facilitating radical resection, its efficacy remains heterogeneous, likely modulated by the intrinsic tumor immune microenvironment (TIME) and therapeutic sequencing (17). Notably, while NIC is associated with a higher incidence of immune-related adverse events (iRAEs), these toxicities are predominantly low-grade and clinically manageable, thereby preserving the favorable benefit-risk profile of this combinatorial approach (18, 19).

Therefore, this study aims to integrate data from existing randomized controlled trials (RCTs) to systematically compare the efficacy and safety of neoadjuvant chemoimmunotherapy and chemotherapy alone in the treatment of ESCC. This analysis will focus on multiple outcome measures, including pathological response, imaging assessment, objective response rate, T-stage changes, and perioperative factors, and will further evaluate the impact of immune-related adverse events on treatment outcomes. By comprehensively analyzing these key factors, the study intends to provide stronger evidence for optimizing neoadjuvant treatment strategies, ultimately improving the therapeutic efficacy and prognosis of ESCC patients.

## Materials and methods

### Search strategy

The keywords used in the search strategy were “neoadjuvant chemotherapy with immunotherapy”, “esophageal squamous cell carcinoma”, and “randomized controlled trial”, Six databases, including PubMed, ScienceDirect, Cochrane Library, Scopus, EMBASE, and Web of Science, were thoroughly searched from their inception to November 5, 2025, as detailed in **Supplementary Table 1**.

### Selection criteria

Inclusion criteria (PICOS) were as follows:

1. Participants (P): ESCC.
2. Intervention (I) and Control (C): NIC (PD-1/PD-L1 inhibitor [such as camrelizumab, toripalimab, or pembrolizumab] in combination with platinum-based chemotherapy) versus NC (platinum-based chemotherapy) alone. Treatment was administered for two to four cycles before surgery, depending on the specific trial protocol.
3. Outcomes (O): Primary outcomes: pCR, and MPR. Secondary outcomes: overall survival (OS), surgery rate, R0 resection rate, AEs, Changes in T Staging, complete response(CR), partial response(PR), stable disease(SD), progressive disease(PD), ORR, and disease control rate(DCR). OS was defined as the time from randomization to death from any cause; CR was defined as the disappearance of all detectable signs of the tumor as assessed by imaging or clinical examination, with no evidence of disease progression; PR was defined as a reduction of at least 30% in the sum of the longest diameters of target lesions, taking the baseline measurements as reference, with no evidence of new lesions or progression; SD was defined as a condition where the tumor does not meet the criteria for either partial response or progression, indicating that the tumor size remains relatively unchanged from baseline; PD was defined as an increase of at least 20% in the sum of the longest diameters of target lesions compared to the smallest sum recorded, or the appearance of new lesions, indicating tumor progression; ORR was defined as the proportion of patients who achieve a CR or PR to the treatment, as assessed by predefined criteria; DCR was defined as the proportion of patients who achieve a CR, PR, or SD, reflecting the overall control of the tumor during treatment. Radiologic response outcomes (CR, PR, SD, PD, ORR, and DCR) were assessed according to RECIST 1.1 criteria reported in the original trials.
4. Study design (S): RCTs. The exclusion criteria included animal studies, letters, news, review articles, meta-analyses, and case reports.

### Data extraction

Two researchers independently collected details on study attributes (e.g., phase, duration), patient demographics (e.g., age, sex), survival outcomes (e.g., OS), pathological response rates (e.g., MPR, pCR), Adverse Events(AEs), and other relevant information. For incomplete data, corresponding authors were contacted for clarification. Any disagreements were resolved through additional review.

### Quality assessment

The quality of the included studies was evaluated using the Cochrane Risk of Bias Assessment Tool.To assess the robustness of the findings, the GRADE (Grading of Recommendations, Assessment, Development and Evaluations) framework was utilized, categorizing the overall certainty of the evidence into four levels: high, moderate, low, and very low.

### Statistical analysis

For overall survival(OS), hazard ratios (HRs) and corresponding 95% confidence intervals were extracted directly from the original studies and pooled for meta-analysis. mean differences (MD) for continuous variables, and Risk ratios (RR) or Odds ratios(OR) for dichotomous variables. A fixed-effects model was applied when the I^2^ statistic was less than 50% or the P-value exceeded 0.1, indicating low heterogeneity; in contrast, a random-effects model was used when heterogeneity was higher. For major outcomes with substantial heterogeneity (I^2^ > 50%), subgroup analyses were undertaken, with sensitivity analyses conducted where indicated, to explore potential sources. Statistical significance was set at a P-value of less than 0.05. Publication bias was evaluated through visual inspection of funnel plots. Data analysis was performed using Review Manager 5.4 and STATA 12.0. The study followed the PRISMA guidelines and was registered with PROSPERO (ID: CRD420251184537).

## Results

### Search results

**Figure 1:**
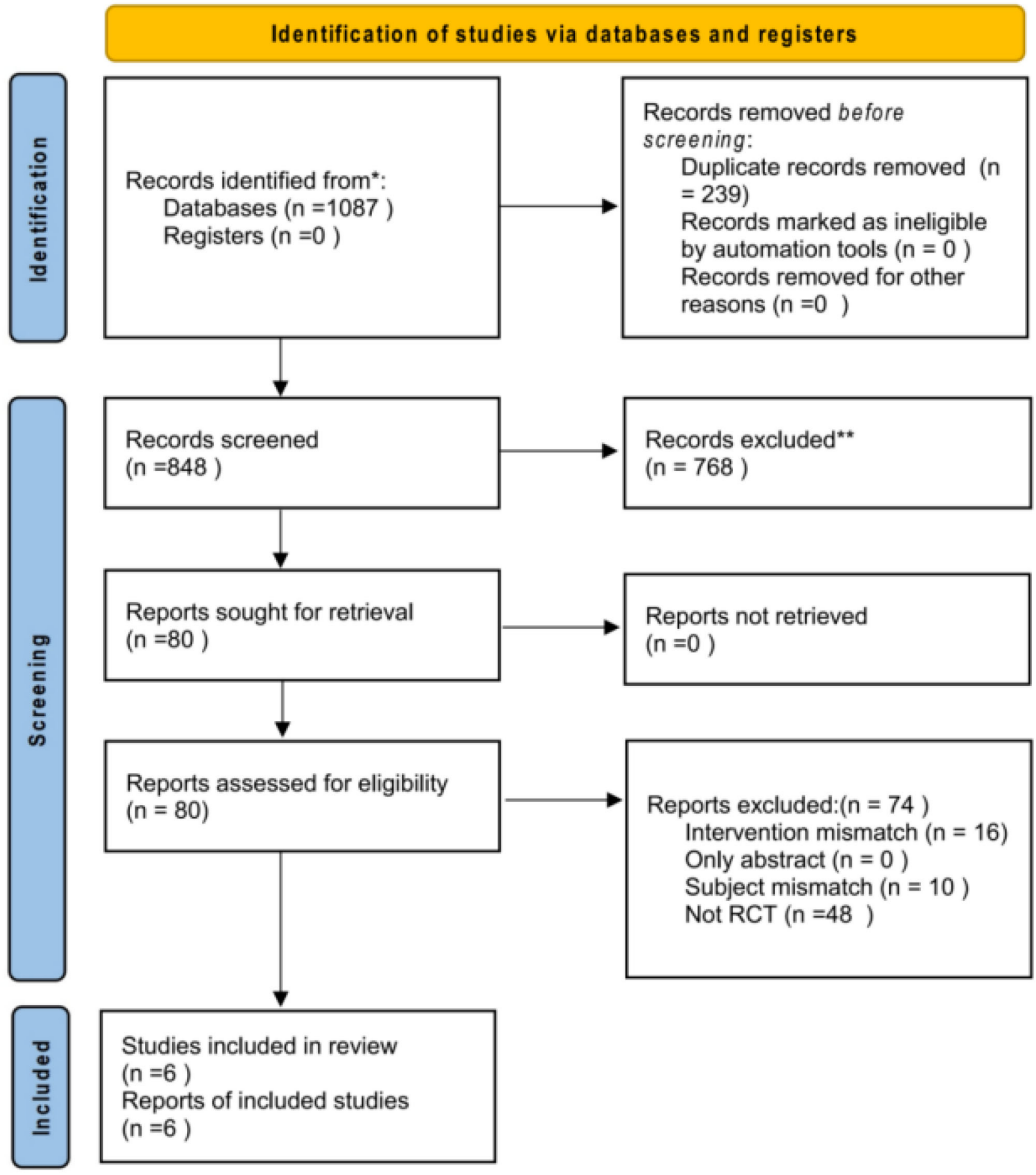
PRISMA.

Among the 1087 studies screened, 6 RCTs comprising 1070 patients were ultimately included (**Figure 1**). All included studies were assessed as high quality (**Supplementary Figures 1**, **2**). The baseline characteristics of these RCTs are summarized in **Table 1**. The certainty of evidence for all major outcomes was evaluated using the GRADE approach, and the Summary of Findings is presented in **Table 2**, number of included studies varied by outcome according to data availability.

**Table 1:** Baseline characteristics.

Study	Phase	Year	Disease stage	Primary endpoint	Groups	Sample size	Patients	Sex (M/F)	Age (mean, year)	ECOG PS 0	ECOG PS 1	ECOG PS 2	ICIs type	Chemotherapy
Jiao (20)	II	2025	T1N2M0 or T2–T4aN1–N2M0	pCR	NIC	90	60	50, 10	62	6	54	0	Nivolumab	Paclitaxel+Cisplatin
					NC		30	26,4	65	1	29	0	-	
Li (21)	II	2023	T2 N1–N3M0 or T3–T4aN1–N3M0	MPR	NIC	64	32	51, 13	62	25	7	0	Socazolimab	Nab-paclitaxel+Cisplatin
					NC		32			27	5	0	-	
Qin (16)	III	2024	T1b-3N1-N3M0 or T3N0M0	pCR	NIC	391	262	228,34	63	106	24	0	Camrelizumab	Paclitaxel+Cisplatin
					NC		129	104,25	65	104	25	0	-	
Wang (22)	II	2023	T2N0-N1M0 or T3~4aN1~2M0	Tumor response assessed by RECIST 1.1	NIC	30	15	13,2	61	15	0	0	Camrelizumab	Docetaxel+Cisplatin+Fluorouracil
					NC		15	15,0	63	15	0	0	-	
Zhang (23)	III	2023	cT1-T4N1-N3M0 or cT3-T4N0M0	pCR and 5-years OS	NIC	150	90	128,22	65	22	68	0	Camrelizumab	Nab-paclitaxel+Cisplatin
					NC		60			14	46	0	-	
Zheng (15)	III	2024	T1N1-N3M0 or T2-3N0-3M0	EFS	NIC	252	127	97,30	66	98	27	2	Toripalimab	Paclitaxel+Cisplatin
					NC		125	97,28	68	105	20	0	-	

ECOG PS, Eastern Cooperative Oncology Group Performance Status; M/F, Male/Female; NC, Neoadjuvant chemotherapy; NIC, Neoadjuvant chemotherapy with PD-1/PD-L1 inhibitors; ICIs, Immune checkpoint inhibitors; MPR, Major pathological response; pCR, Pathological complete response; RECIST, Response evaluation criteria in solid tumors; EFS, Event-free survival; OS, Overall survival.

Author(s): yibang Ye.

Question: Neoadjuvant Chemotherapy with Immunotherapy compared to Neoadjuvant Chemotherapy for Esophageal Squamous Cell Carcinoma.

Setting:. Bibliography: .

**Table 2:** GRADE assessment of evidence for major outcomes.

Certainty assessment							№ of patients		Effect		Certainty	Importance
№ of studies	Study design	Risk of bias	Inconsistency	Indirectness	Imprecision	Other considerations	Neoadjuvant chemotherapy with immunotherapy	Neoadjuvant chemotherapy	Relative (95% CI)	Absolute (95% CI)		
MPR												
6	randomised trials	not serious	not serious	not serious	not serious	none	239/558 (42.8%)	78/351 (22.2%)	**OR 2.40** (1.45 to 3.98)	**185 more per 1,000** (from 71 more to 310 more)	#x2A01;#x2A01;#x2A01;#x2A01; High	CRITICAL
pCR												
6	randomised trials	not serious	not serious	not serious	not serious	none	124/558 (22.2%)	28/351 (8.0%)	**OR 3.53** (2.26 to 5.53)	**155 more per 1,000** (from 84 more to 244 more)	#x2A01;#x2A01;#x2A01;#x2A01; High	CRITICAL
OS												
2	randomised trials	not serious	not serious	not serious	serious^a^	none	-/187	-/155	**HR 0.57** (0.32 to 1.01)	**-- per 1,000** (from -- to --)	#x2A01;#x2A01;#x2A01;◯ Moderate^a^	CRITICAL
EFS												
2	randomised trials	not serious	not serious	not serious	serious^a^	none	-/187	-/155	**HR 0.72** (0.49 to 1.06)	**-- per 1,000** (from -- to --)	#x2A01;#x2A01;#x2A01;◯ Moderate^a^	CRITICAL
R0 resection												
4	randomised trials	not serious	not serious	not serious	not serious	none	408/416 (98.1%)	240/250 (96.0%)	**OR 2.56** (1.01 to 6.50)	**24 more per 1,000** (from 0 fewer to 34 more)	#x2A01;#x2A01;#x2A01;#x2A01; High	CRITICAL
Surgery rate												
4	randomised trials	not serious	not serious	not serious	not serious	none	416/481 (86.5%)	250/316 (79.1%)	**OR 1.57** (1.07 to 2.31)	**65 more per 1,000** (from 11 more to 106 more)	#x2A01;#x2A01;#x2A01;#x2A01; High	CRITICAL
Time from last neoadjuvant dose to definitive surgery												
4	randomised trials	not serious	very serious^b^	not serious	not serious	none	416	250	–	MD **4.73 higher** (1.56 higher to 7.9 higher)	#x2A01;#x2A01;◯◯ Low^b^	IMPORTANT
The number of lymph nodes resected												
4	randomised trials	not serious	not serious	not serious	not serious	none	416	250	–	MD **1.76 higher** (0.74 higher to 2.79 higher)	#x2A01;#x2A01;#x2A01;#x2A01; High	IMPORTANT
T staging												
2	randomised trials	not serious	not serious	not serious	serious^a^	none	77/252 (30.6%)	59/177 (33.3%)	**OR 0.84** (0.51 to 1.39)	**38 fewer per 1,000** (from 130 fewer to 77 more)	#x2A01;#x2A01;#x2A01;◯ Moderate^a^	IMPORTANT
Total AEs												
4	randomised trials	not serious	serious^c^	not serious	not serious	none	444/474 (93.7%)	295/316 (93.4%)	**RR 1.03** (0.99 to 1.07)	**28 more per 1,000** (from 9 fewer to 65 more)	#x2A01;#x2A01;#x2A01;◯ Moderate^c^	CRITICAL
Grade 3–4 AEs												
5	randomised trials	not serious	not serious	not serious	not serious	none	124/519 (23.9%)	74/361 (20.5%)	**RR 1.10** (0.87 to 1.40)	**20 more per 1,000** (from 27 fewer to 82 more)	#x2A01;#x2A01;#x2A01;#x2A01; High	CRITICAL
iRAEs												
4	randomised trials	not serious	serious^d^	not serious	not serious	none	110/474 (23.2%)	4/346 (1.2%)	**RR 16.92** (6.55 to 43.75)	**184 more per 1,000** (from 64 more to 494 more)	#x2A01;#x2A01;#x2A01;◯ Moderate^d^	CRITICAL
Serious AEs												
4	randomised trials	not serious	very serious^e^	not serious	not serious	none	421/474 (88.8%)	267/316 (84.5%)	**RR 1.05** (1.00 to 1.10)	**42 more per 1,000** (from 0 fewer to 84 more)	#x2A01;#x2A01;◯◯ Low^e^	CRITICAL
AEs leading to discontinuation												
3	randomised trials	not serious	not serious	not serious	not serious	none	11/414 (2.7%)	5/286 (1.7%)	**RR 1.45** (0.48 to 4.44)	**8 more per 1,000** (from 9 fewer to 60 more)	#x2A01;#x2A01;#x2A01;#x2A01; High	CRITICAL

CI, confidence interval; HR, hazard ratio; MD, mean difference; OR, odds ratio; RR, risk ratio. Bold values indicate the pooled point estimate of the effect (with 95% CI) derived from meta-analysis.

Explanations.

^a^ Downgraded due to imprecision (limited number of studies.

^b^ I^2^ = 95%.

^c^ I^2^ = 77%.

^d^ I^2^ = 76%.

^e^ I^2^ = 92%.

### Pathological responses

**Figure 2:**
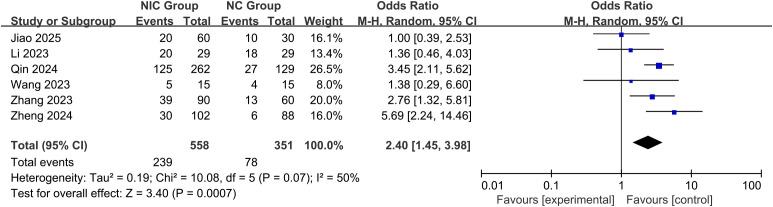
Forest plots of MPR rates and pCR rates between the NIC and NC.

The MPR rate was significantly higher in the NIC group than in the NC group (42.8% vs. 22.2%; OR = 2.40[1.45–3.98], P = 0.0007). Similarly, the pCR rate was also significantly increased in the NIC group (22.2% vs. 8.0%; OR = 3.53[2.26–5.53], P < 0.00001) (**Figure 2**).

### Survival

**Figure 3:**
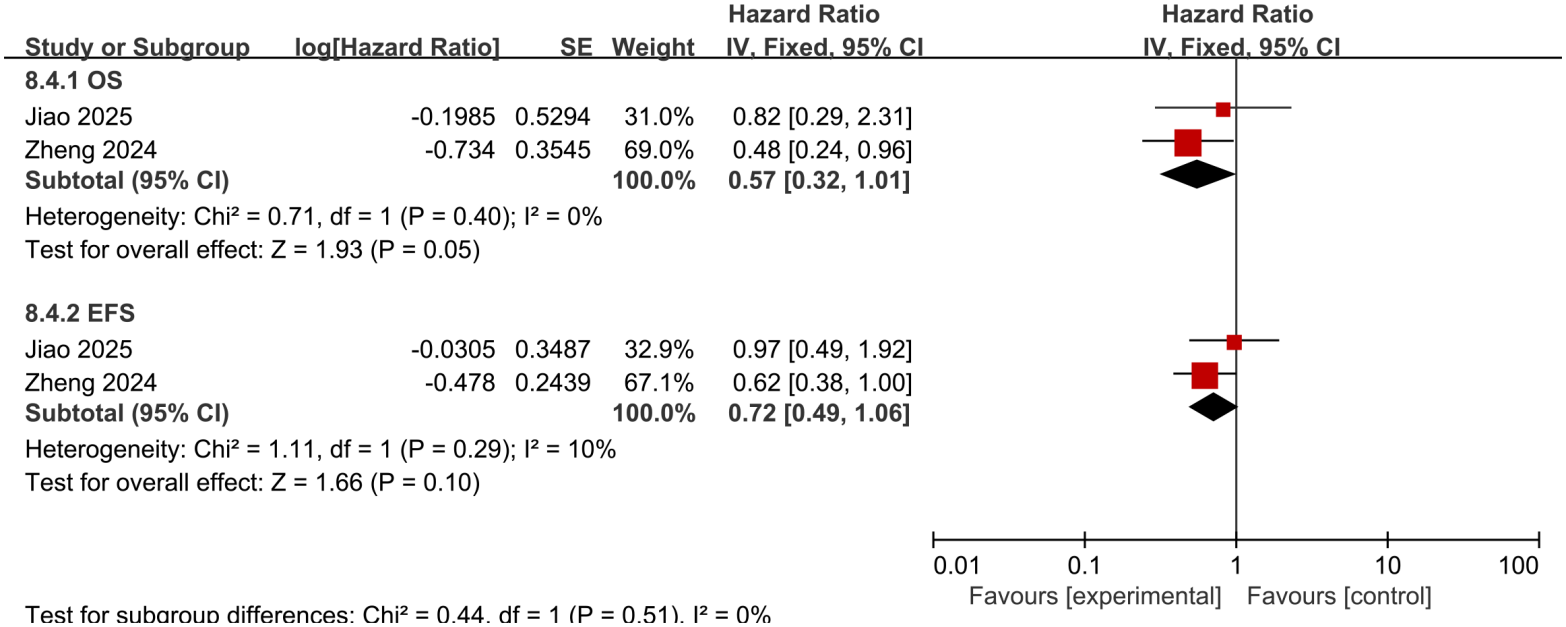
Forest plot of HRs for overall survival (OS) and event-free survival (EFS) between the NIC and NC.

Two randomized controlled trials (Jiao 2025 and Zheng 2024) reported HR of overall survival (OS) and event-free survival (EFS). For OS, pooled analysis favored neoadjuvant immunochemotherapy (NIC) over neoadjuvant chemotherapy (NC), with borderline significance (HR = 0.57[0.32–1.01], p = 0.05). However, OS should be interpreted cautiously because only two RCTs reported survival outcomes and follow-up was limited. For EFS, the pooled result also favored NIC, but the difference was not significant (HR = 0.72, [0.49–1.06], p = 0.10) (**Figure 3**).

### Surgery

**Figure 4:**
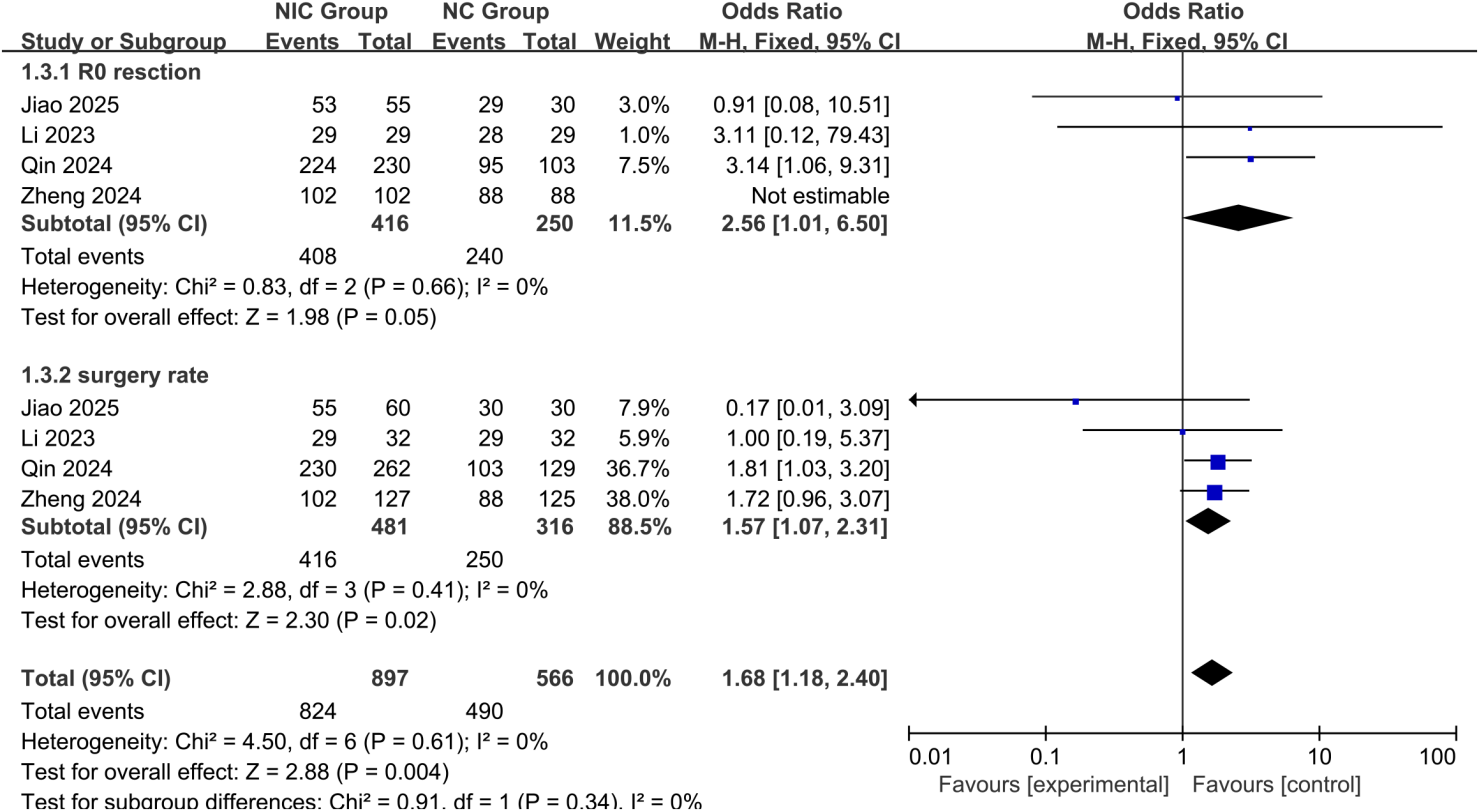
Forest plots of R0 resection and surgery rate between the NIC and NC.

The NIC group demonstrated a significantly higher surgery rate compared to the NC group (OR: 1.57, [1.07–2.31]; P = 0.02), indicating better surgical accessibility or feasibility in terms of overall procedure rates. Moreover, The difference in R0 resection rate between the two groups reached borderline statistical significance (OR: 2.56, [1.01–6.50]; P = 0.05) (**Figure 4**).

### Intraoperative and hospitalization indicators

**Figure 5:**
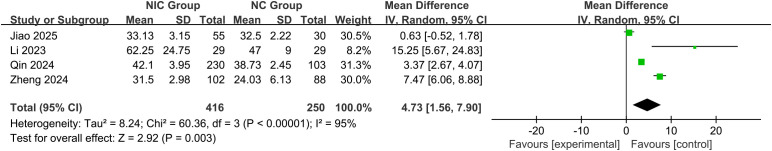
Forest plots of time from last neoadjuvant dose to definitive surgery.

**Figure 6:**
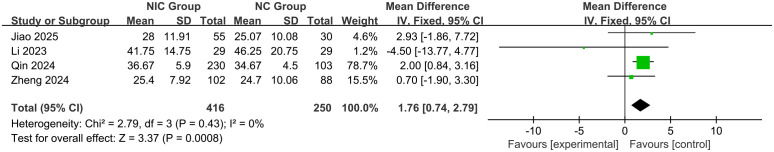
Forest plots of the number of lymph nodes resected.

A significantly longer interval from the last neoadjuvant treatment to definitive surgery was observed in the NIC group compared with the NC group (MD: 4.73[1.56–7.90]; P = 0.003) (**Figure 5**). However, caution is warranted (I^2^ = 95%). No effect modification by phase (P = 0.82), but significant modification by treatment duration (P = 0.03), favoring greater prolongation with 3–4 versus 2 cycles (**Supplementary Table 2**). In addition, the number of lymph nodes resected during surgery was significantly higher in the NIC group than in the NC group (MD: 1.76[0.74–2.79]; P = 0.0008). (**Figure 6**).

In contrast, no statistically significant differences were observed between the two groups in terms of operative duration, intraoperative blood loss, or length of postoperative hospital stay (**Supplementary Figure 3**).

### T staging

**Figure 7:**
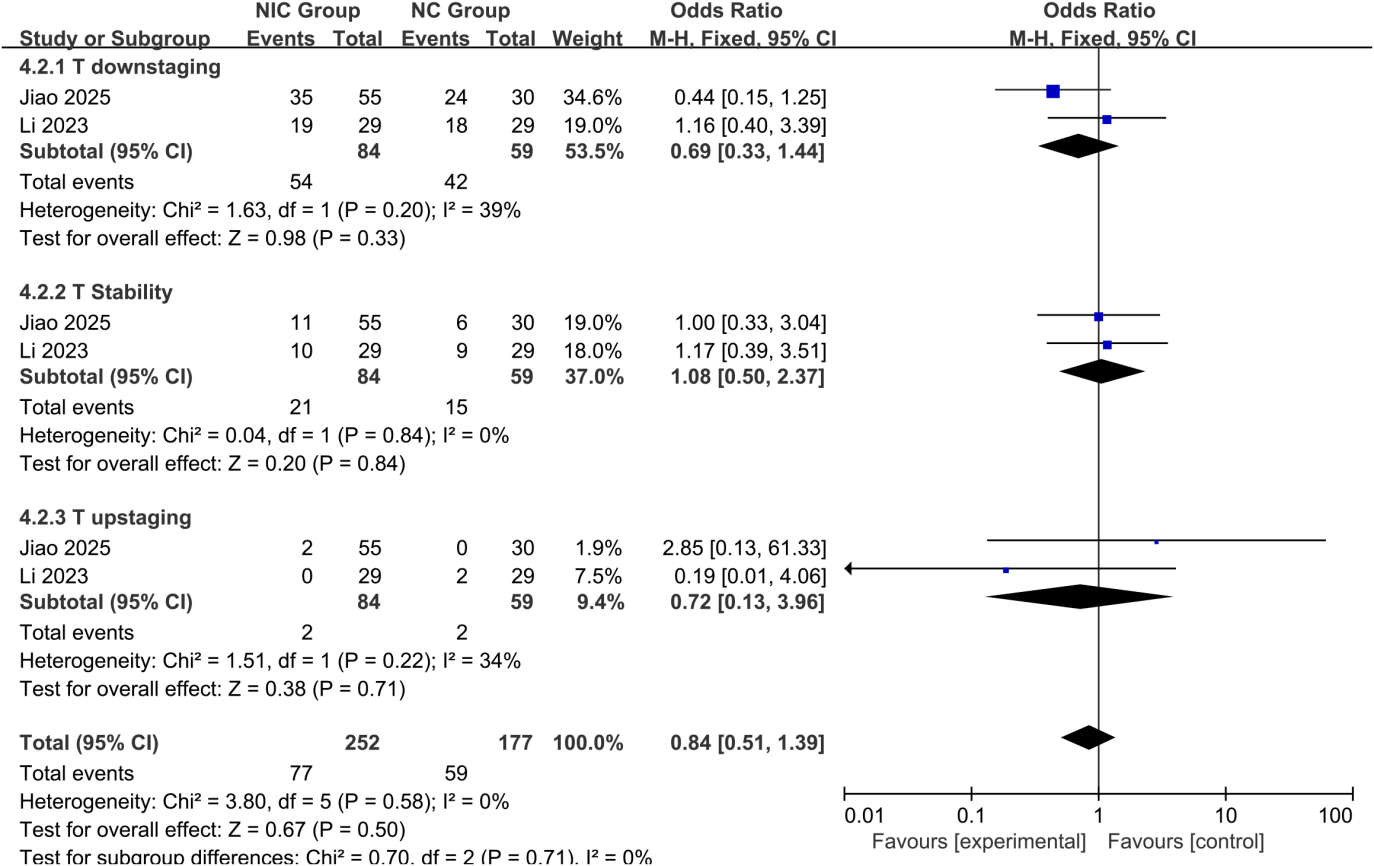
Forest plots of T staging.

No significant statistical differences were observed between the NIC and NC groups regarding changes in T-stage, including downstaging, stability, and upstaging, as assessed by both clinical T-stage (ycT) and pathological T-stage (ypT) (**Figure 7**).

### Response evaluation criteria in solid tumors

Based on RECIST criteria, pooled fixed-effects analyses showed no statistically significant differences between the NIC and NC groups in complete response (CR) (RR = 5.00 [0.26–96.13], p = 0.29), partial response (PR) (RR = 1.03[0.84–1.25], p = 0.80), stable disease (SD) (RR = 0.73[0.44–1.21], p = 0.23), or progressive disease (PD) (RR = 0.82[0.23–2.97], p = 0.76). Similarly, no significant differences were observed in objective response rate (ORR) (RR = 1.75 [0.64–4.75], p = 0.27) or disease control rate (DCR) (RR = 1.08[0.85–1.37]; p = 0.55). (**Supplementary Figure 4**).

### Safety

In the safety analysis, no significant differences were observed between the(NIC) and neoadjuvant chemotherapy (NC) groups in total adverse events (AEs) (93.67% vs. 93.35%, RR = 1.03[0.99–1.07], p=0.19), grade 3–4 AEs (40.33% vs. 20.50%, RR = 1.10[0.87–1.40], p=0.41), serious AEs (88.82% vs. 84.49%, RR = 1.05[1.00–1.10]m, p=0.06), or AEs leading to treatment discontinuation (2.66% vs. 1.75%, RR = 1.45[0.48–4.44], p = 0.51). In contrast, immune-related adverse events (iRAEs) were significantly more frequent in the NIC group than in the NC group (23.21% vs. 1.16%, RR = 16.92[6.55–43.75, p<0.00001) (**Table 3**).

**Table 3:** Comparison of adverse events between the NIC and NC groups.

Adverse events	NIC		NC		Risk ratio [95% CI]	P
	Event/total	%	Event/total	%		
Total AEs	444/474	93.67	295/316	93.35	1.03 [0.99, 1.07]	P = 0.19
Grade 3–4 AEs	124/519	40.33	74/361	20.50	1.10 [0.87, 1.40]	P = 0.41
iRAEs	110/474	23.21	4/346	1.16	16.92 [6.55, 43.75]	P<0.00001
Serious AEs	421/474	88.82	267/316	84.49	1.05 [1.00, 1.10]	P = 0.06
AEs leading to discontinuation	11/414	2.66	5/286	1.75	1.45 [0.48, 4.44]	P = 0.51

AEs, Adverse Events; iRAEs, Immune-Related Adverse Events.

Subgroup analyses showed that neither study phase nor treatment duration significantly modified the estimates for immune-related adverse events (iRAEs), serious adverse events (AEs), or total AEs (all p for subgroup difference > 0.05) (**Supplementary Table 3**).

In the analysis of all-grade adverse events, significant between-group differences(p<0.05) were observed for fatigue, anemia, leukopenia, neutropenia, thrombocytopenia, anorexia, rash, hyponatremia, and thyroid dysfunction, whereas no significant differences were found for the remaining adverse events. In addition, postoperative surgical and pulmonary complications were comparable between the two groups, with no statistically significant differences identified (**Supplementary Table 4**).

### Publication bias

**Figure 8:**
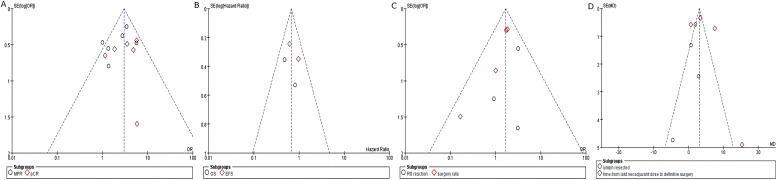
Funnel plots of pathological responses **(A)**, HRs for survival **(B)**, surgery **(C)**, Intraoperative and hospitalization indicators **(D)**.

**Figure 9:**
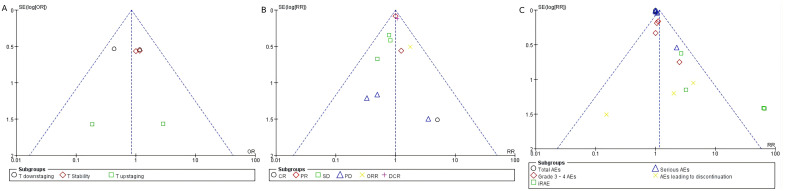
Funnel plots of T staging **(A)**, RECIST **(B)** and safety summary **(C)**.

The funnel plot symmetry for pathological responses, HRs for Survival, surgery rates, Time from the last neoadjuvant treatment to definitive surgery, the number of lymph nodes resected (**Figure 8**), T stage, RECIST and the safety summary suggested an acceptable degree of publication bias (**Figure 9**).

## Discussion

NC has been widely adopted as a neoadjuvant treatment option for resectable ESCC in several clinical settings (24). However, as research has evolved, the limitations of NC in achieving optimal pathological responses and long-term survival outcomes have become more apparent. In contrast, ICIs, particularly those targeting the PD-1/PD-L1 pathway, have demonstrated substantial efficacy in treating ESCC at various stages, sparking increasing interest in their use within neoadjuvant therapy (25, 26). While the incorporation of ICIs into neoadjuvant chemotherapy regimens represents a promising new treatment approach, important questions remain about the additional efficacy, potential toxicities, and the identification of the most appropriate patient populations (19). This meta-analysis consolidates data from RCTs to provide a systematic comparison of the effectiveness and safety of neoadjuvant chemoimmunotherapy (NIC) versus neoadjuvant chemotherapy (NC) in the treatment of resectable ESCC.

Although the included studies differed in ICI agents, chemotherapy regimens, treatment duration, and study phase, additional subgroup and sensitivity analyses did not indicate that these variations materially changed the main pooled results. For MPR and pCR, the treatment effect was directionally consistent across most subgroups, and exclusion of trial (Jiao, 2025) substantially reduced heterogeneity in the paclitaxel-based analyses while confirming the robustness of the overall estimates (**Supplementary Table 5**). Subgroup analyses of surgical timing and adverse-event outcomes yielded comparable overall patterns despite heterogeneity in some comparisons. These results suggest that the pooling strategy was broadly appropriate for clinically related outcome domains, while acknowledging that residual heterogeneity cannot be fully excluded.

Importantly, these findings interpreted in light of evidence certainty. GRADE assessment showed high-certainty evidence for improved MPR, pCR, surgery rate, and R0 resection with NIC, supporting the robustness of its short-term pathological and surgical benefits. By contrast, the evidence for OS and EFS was moderate, and several perioperative or exploratory outcomes were supported by low-to-moderate certainty, owing to limited data and heterogeneity. These results suggest that the clearest current value of NIC lies in enhancing tumor regression and resectability.

In the pathological response analysis of NIC, numerous studies have reported significant improvements in MPR and pCR compared with NC alone. However, concerns remain regarding the robustness and reproducibility of these findings across different clinical settings (27) (14) (28). By integrating data from high-quality studies (21) (23) (22) (16) (20) (15). The present study further confirms the significant advantage of NIC over NC in achieving both MPR and pCR, underscoring the substantial pathological impact of immunotherapy in ESCC.

Notably, substantial heterogeneity was observed in the MPR analysis (I^2^ = 50%), which may be attributable to variations in immune checkpoint inhibitors, chemotherapy regimens, baseline patient characteristics, and study design (14) (29) (11). The differential response patterns of MPR and pCR highlight the multidimensional nature of immunochemotherapeutic effects. Compared with pCR, MPR may more sensitively capture alterations within the tumor microenvironment, whereas pCR, despite being a stringent endpoint, may not fully reflect the long-term immunological benefits of treatment. Moreover, a potential dissociation may exist between early pathological response and the underlying biological effects of immunotherapy, particularly when evaluating immune-mediated durable antitumor activity (30, 31).

Although pCR has been widely adopted as a surrogate endpoint in the context of conventional chemotherapy, its interpretation in the immunotherapy era presents unique challenges. The therapeutic effects of immunotherapy extend beyond preoperative tumor burden reduction to include postoperative immune surveillance and suppression of micrometastatic disease (32). Therefore, future investigations should incorporate multidimensional assessment strategies, including radiological evaluation and dynamic changes in immune-related biomarkers to provide a more comprehensive evaluation of NIC efficacy (33, 34).

In summary, although both pCR and MPR have demonstrated meaningful clinical benefits in contemporary trials, they should not be regarded as standalone definitive endpoints in the context of immunochemotherapy. With the rapid evolution of immunotherapeutic strategies, future research should focus on integrating multiple efficacy endpoints to better characterize the overall therapeutic impact, particularly the potential for long-term survival benefit (35). Furthermore, rigorously designed multicenter randomized controlled trials are warranted to clarify the relationship between pathological response parameters (MPR and pCR) and long-term survival outcomes, thereby providing more precise evidence to guide clinical implementation of immunochemotherapy.

In terms of survival, pooled analysis of the hazard ratios reported in the included trials suggested a potential overall survival(OS) benefit of NIC over NC, although the difference was only of borderline statistical significance (HR = 0.57[0.32–1.01], p=0.05). No significant difference was observed in event-free survival(EFS) (HR = 0.72, [0.49–1.06], p= 0.10). These findings suggest that the potential survival benefit of NIC is biologically plausible and may reflect an important shift in the role of neoadjuvant therapy for ESCC. Chemotherapy may debulk the tumor and promote antigen exposure, while immune checkpoint inhibition can restore antitumor immune responsiveness (36, 37). In NIC, this combination takes advantage of the intact tumor as an *in situ* source of antigen, potentially generating a more comprehensive and durable immune response (38). Although the updated analysis showed only borderline significance for OS and no significant difference in EFS, the observed trend remains clinically meaningful. It is possible that the benefit of NIC is not fully captured by conventional short-term efficacy indicators, but may instead stem from modulation of the tumor–immune microenvironment with potential implications for long-term disease control (39, 40).

Although NIC tended to improve OS/EFS versus NC, survival evidence remains inconclusive because only two RCTs were available and significance was borderline. Further long-term follow-up and phase III RCTs are needed.

Another important consideration in interpreting survival outcomes is that postoperative adjuvant treatment was not reported in a sufficiently uniform or extractable manner across the included studies. As a result, a formal subgroup analysis according to adjuvant therapy use was not feasible, and whether survival differed based on adjuvant therapy use could not be reliably determined in the present meta-analysis.

Our analysis showed that NIC significantly improved the overall surgery rate (P = 0.02), and was associated with a borderline increase in the R0 resection rate(OR: 2.56, [1.01–6.50]; P = 0.05). This pattern suggests that the benefit of NIC may lie not only in facilitating resection, but also in improving local tumor control and limiting occult micrometastatic disease (41). Whereas surgery rate reflects operability, R0 resection is a more stringent indicator of surgical radicality and likely depends on multiple factors beyond technical feasibility, including tumor extent, immune context, treatment timing, and underlying tumor biology (11). Selected toxicities may still impair perioperative fitness or delay surgery, thereby influencing both resection and surgical quality (42). The borderline significance for R0 resection may therefore reflect the interplay of treatment heterogeneity, biological variation, and differences in host immune responsiveness (16) (20). More broadly, these findings support a growing view that NIC may contribute not only to increasing surgical access, but also to redefining the goals of neoadjuvant therapy in ESCC. Future research should emphasize the long-term evaluation of treatment responses, and further refine the quality of surgical resection.

This study reveals distinct perioperative characteristics of neoadjuvant immunochemotherapy compared to traditional treatments. No significant differences were observed between the NIC and NC groups in intraoperative blood loss, surgery duration, or postoperative hospitalization, indicating that immunotherapy does not increase surgical complexity or perioperative burden (43). The NIC group had a significantly longer therapy-to-surgery interval and a higher number of resected lymph nodes, These findings may suggest that neoadjuvant immunochemotherapy influences perioperative patterns beyond conventional measures of surgical difficulty.

The longer interval from the last neoadjuvant treatment to definitive surgery suggests that immune-mediated antitumor effects require time. While surgery duration and blood loss were unchanged, increased lymph node clearance indicates that immunochemotherapy alters the biological landscape, making regional lesions more detectable and easier to remove (44).

However, The prolonged interval from the last neoadjuvant treatment to definitive surgery showed substantial heterogeneity, likely driven by treatment duration, other protocols and center-level differences. Thus, these findings should be interpreted cautiously. Our subgroup analysis suggests that this variability is more likely related to treatment duration than to study phase, with more cycles associated with a greater delay to surgery. This pattern may reflect cumulative treatment exposure and perioperative recovery demands rather than a trial-phase effect, underscoring the importance of balancing regimen intensity against operative readiness and of standardizing perioperative pathways across studies (45).

Traditionally, researches have focused on surgical difficulty and risk, parameters like the interval from the last neoadjuvant treatment to definitive surgery and lymph node resection may better reflect tumor biology in the context of neoadjuvant immunotherapy. A purely technical interpretation may overlook immunotherapy’s biological effects during the neoadjuvant phase (46, 47).

In conclusion, this study broadens the scope of perioperative outcome interpretation, emphasizing the need to integrate these outcomes with pathological responses and long-term survival data to fully assess the effects of NIC.

Regarding T-stage changes, In most studies, the extent of T-stage downstaging was minimal, suggesting that while NIC improved local disease control, its impact on T-stage was limited (20, 21). The effects of NIC may be more noticeable in the modulation of the tumor microenvironment and the immune clearance of residual tumor, rather than in significant changes to T-stage (47). It is essential to recognize that changes in T-stage are influenced by several factors, such as tumor size, lymph node involvement, and the patient’s immune status (48). Therefore, the absence of significant T-stage changes with NIC treatment does not imply treatment failure; rather, it may reflect the deeper immunomodulatory effects of immunotherapy on the tumor microenvironment (49). Additionally, T-stage downstaging does not necessarily correlate directly with postoperative prognosis. Long-term follow-up and biomarker analysis are crucial to further understanding the relationship between T-stage and patient prognosis. Future large-scale studies are needed to verify this relationship.

In RECIST-based studies, no significant differences were observed between the NIC and NC groups in ORR, CR, PR, PD, SD, or DCR. This suggests that the addition of immunotherapy did not show a clear advantage in radiological response based on conventional response criteria.

The RECIST system has limitations in immunotherapy contexts. Its traditional reliance on tumor volume reduction suits cytotoxic therapies (50, 51), while immunotherapy works through tumor microenvironment modulation and immune surveillance restoration, effects not immediately reflected in radiological changes (52, 53). Therefore, the absence of significant differences in ORR, CR, or PR may reflect the limited sensitivity of RECIST in capturing the full spectrum of immune-related therapeutic effects, rather than the absence of clinical value.

In addition, the limited number of included studies and the wide confidence intervals of several endpoints suggest that these analyses may have been underpowered. However, for long-term survival prediction, further validation with pathological response and survival data is needed (54). This study emphasizes that RECIST-based outcomes are useful but should not be isolated as final efficacy measures in neoadjuvant immunochemotherapy.

From a clinical perspective, efficacy assessments should move beyond volumetric criteria, integrating dynamic imaging, circulating tumor DNA, immune infiltration, and pathological responses to align with the biological mechanisms of immunotherapy (55, 56). This approach will enhance the accuracy of immunochemotherapy benefit assessments and provide a solid basis for patient stratification and treatment decisions.

In terms of safety, no significant differences were observed between the NIC and NC groups in the overall incidence of total adverse events (AEs), grade 3–4 AEs, serious AEs, or AEs leading to treatment discontinuation, indicating that neoadjuvant immunochemotherapy did not effectively reduce severe toxicity risks compared to conventional chemotherapy. In this context, safety should be interpreted not merely in terms of event frequency, but also in light of how toxicity profiles affect treatment tolerability, maintenance of therapeutic intensity, and the overall risk–benefit balance (57).

In contrast, the incidence of immune-related adverse events (iRAEs) was significantly higher in the NIC group than in the NC group (23.21% vs. 1.16%, P < 0.0001), which is consistent with the mechanism of immune checkpoint inhibition and reflects treatment-related immune activation. While iRAEs are conventionally regarded as adverse events, they may also serve as biological indicators of immune engagement in the neoadjuvant setting (58), although their relationship with pathological response and long-term outcomes still requires further prospective validation.

Notably, although no statistically significant difference was identified in serious AEs(p=0.06) after the analysis, the numerical increase observed in the NIC group suggests that careful monitoring remains necessary, particularly when immune-related toxicities may alter treatment delivery or perioperative management. Thus, the safety profile of NIC appears to be characterized less by a generalized increase in toxicity and more by a shift in toxicity spectrum related to immune activation, which may have implications for treatment planning and supportive care (59). Therefore, when developing neoadjuvant immunochemotherapy strategies, greater attention should be paid to the identification, monitoring, and management of immune-related toxicities, rather than relying solely on conventional summary measures of adverse events (60).

In addition, subgroup analyses did not show significant modification of total AEs, serious AEs, or iRAEs by study phase or treatment duration, suggesting that the overall safety pattern was relatively consistent across the available study settings. This finding should still be interpreted cautiously, given the limited number of studies and the restricted power of subgroup comparisons. At the event-specific level, several all-grade adverse events differed significantly between groups, whereas postoperative surgical and pulmonary complications were comparable, indicating that NIC may change the composition of treatment-related toxicity without clearly increasing postoperative risk.

This study provides an updated synthesis of RCT evidence comparing neoadjuvant chemoimmunotherapy with chemotherapy alone in ESCC patients, offering a more comprehensive assessment of efficacy and safety and informing the optimized management of neoadjuvant immunochemotherapy. Future research should further clarify the links between immune-related toxicities, antitumor immune responses, and predictive biomarkers to improve quality of life and long-term survival.

A limitation of this study is that, while the total sample size of the included RCTs was large (n = 1070), Egger’s test was not performed due to the limited number of studies (<10) (61). Furthermore, the differences in study design and the heterogeneity in follow-up durations across studies may impact the consistency of the conclusions. Specifically, the short follow-up periods and the variability in immunotherapy regimens contributed to some heterogeneity in the results (I^2^ > 50%). Notably, some perioperative outcomes, particularly time to surgery, showed substantial heterogeneity, likely related to treatment duration, other protocols or center-level differences. Residual clinical heterogeneity across studies remains a limitation, particularly regarding ICI agents, chemotherapy regimens, and incompletely reported treatment cycles. Moreover, subgroup analyses were constrained by the small number of studies, precluding some clinically relevant comparisons that were either statistically underpowered or methodologically infeasible. Additionally, the absence of individualized patient data restricted the ability to conduct detailed subgroup analyses based on baseline immune markers and tumor characteristics, limiting our understanding of how different patient groups respond to treatment. Future studies should focus on standardizing immunotherapy protocols, improving patient stratification, and collecting long-term survival data to provide a more comprehensive assessment of the long-term efficacy of NIC.

## Conclusion

NIC appears to offer systemic therapeutic benefits by enhancing tumor immunogenicity and promoting immune surveillance. However, the partial high heterogeneity observed in this study indicates that NIC’s efficacy is influenced by factors such as baseline patient characteristics, treatment regimens, and the management of immune-related adverse events. Future resea0rch should focus on the standardization of treatment protocols, stratified therapy approaches, and further investigation into the long-term impact of immune-related adverse events on treatment outcomes. Additionally, future randomized controlled trials (RCTs) should integrate more long-term survival data and improved immune biomarker screening to better assess treatment efficacy.

## Supplementary material

Supplementary Figure 3 Forest plots of hospital intervention.

Supplementary Figure 4 Forest plots of RECIST.

## Glossary

ESCC: Esophageal squamous cell carcinoma
NC: Neoadjuvant chemotherapy
NIC: Neoadjuvant chemotherapy with PD-1/PD-L1 inhibitors
ICIs: Immune checkpoint inhibitors
CR: Complete response
PR: Partial response
SD: Stable disease
PD: Progressive disease
ORR: Objective response rate
DCR: Disease control rate
R0 resection: microscopically margin-negative resection
PD-1: Programmed death-1
PD-L1: Programmed death-ligand 1
AE: Adverse Events
pCR: Pathological complete response
PICOS:Participants: Interventions, Comparators, Outcomes, Study design
OS: Overall survival
EFS: Event-free survival
MPR: Major pathological response
IrAE: Immune-related adverse event
RECIST: Response evaluation criteria in solid tumors
HR: Hazard ratio
RR: Risk ratio
GRADE: Grading of Recommendations Assessment, Development and Evaluation
MD: Mean difference
PRISMA: Preferred Reporting Items for Systematic Reviews and Meta-Analyses
PROSPERO: International Prospective Register of Systematic Reviews Randomized controlled trial
RCT: Randomized controlled trial
RR: Risk ratio
TMB: Tumor mutational burden
TRAE: Treatment-related adverse event
iRAEs: immune-related adverse events.
Adverse Events: NIC
Event/Total: %Total AEs, 444/474
Grade 3–4 AEs: 124/519
iRAEs: 110/474
Serious AEs: 421/474
AEs leading to discontinuation: 11/414.
