# Supplementary material for: Efficacy and safety of neoadjuvant chemotherapy with immunotherapy versus chemotherapy alone in esophageal squamous cell carcinoma: a meta-analysis based on randomized controlled trials
Source: Front Immunol. 2026 Jul 9;17:1825905. doi: 10.3389/fimmu.2026.1825905 (PMC13391947; doi:10.3389/fimmu.2026.1825905)
Supplement: Supplementary file 5 [file Table1.docx]

**Table S1** Search strategy.

| **PubMed**  The database was searched on November 5, 2025, n=84.  Search Strategy:  **#1 Search: (((((((((((((((((Esophageal Neoplasms[MeSH Terms]) OR (Esophageal Neoplasm[Title/Abstract])) OR (Neoplasm, Esophageal[Title/Abstract])) OR (Esophagus Neoplasm[Title/Abstract])) OR (Esophagus Neoplasms[Title/Abstract])) OR (Neoplasm, Esophagus[Title/Abstract])) OR (Neoplasms, Esophagus[Title/Abstract])) OR (Neoplasms, Esophageal[Title/Abstract])) OR (Cancer of Esophagus[Title/Abstract])) OR (Esophageal Cancer[Title/Abstract])) OR (Cancer, Esophageal[Title/Abstract])) OR (Cancers, Esophageal[Title/Abstract])) OR (Esophageal Cancers[Title/Abstract])) OR (Cancer of the Esophagus[Title/Abstract])) OR (Esophagus Cancer[Title/Abstract])) OR (Cancer, Esophagus[Title/Abstract])) OR (Cancers, Esophagus[Title/Abstract])) OR (Esophagus Cancers[Title/Abstract])Sort by: Most Recent n = 9617**  **#2 Search: (((((((((((((Neoadjuvant Therapy[MeSH Terms]) OR (Neoadjuvant Therapies[Title/Abstract])) OR (Therapy, Neoadjuvant[Title/Abstract])) OR (Neoadjuvant Treatment[Title/Abstract])) OR (Neoadjuvant Treatments[Title/Abstract])) OR (Treatment, Neoadjuvant[Title/Abstract])) OR (Neoadjuvant Chemotherapy[Title/Abstract])) OR (Chemotherapy, Neoadjuvant[Title/Abstract])) OR (Neoadjuvant Chemotherapies[Title/Abstract])) OR (Neoadjuvant Chemotherapy Treatment[Title/Abstract])) OR (Chemotherapy Treatment, Neoadjuvant[Title/Abstract])) OR (Neoadjuvant Chemotherapy Treatments[Title/Abstract])) OR (Treatment, Neoadjuvant Chemotherapy[Title/Abstract])) Sort by: Most Recent n = 9155**  **#3 Search: (((((Immune Checkpoint Inhibitors[MeSH Terms]) OR (Checkpoint Inhibitors, Immune[Title/Abstract])) OR (Immune Checkpoint Blockers[Title/Abstract])) OR (Checkpoint Blockers, Immune[Title/Abstract])) OR (Immune Checkpoint Inhibitor[Title/Abstract])) OR (Checkpoint Inhibitor, Immune[Title/Abstract]) n = 8107**  **#1 and #2 and #3 n = 84** |
| --- |
| **Web of Science**  The database was searched on November 5, 2025, n=48  Search Strategy:  ((TS=("Neoadjuvant Therapy" OR "Neoadjuvant Therapies" OR "Therapy, Neoadjuvant" OR "Neoadjuvant Treatment" OR "Neoadjuvant Treatments" OR "Treatment, Neoadjuvant" OR "Neoadjuvant Chemotherapy" OR "Chemotherapy, Neoadjuvant" OR "Neoadjuvant Chemotherapies")) OR (TI=("Neoadjuvant Therapy" OR "Neoadjuvant Therapies" OR "Therapy, Neoadjuvant" OR "Neoadjuvant Treatment" OR "Neoadjuvant Treatments" OR "Treatment, Neoadjuvant" OR "Neoadjuvant Chemotherapy" OR "Chemotherapy, Neoadjuvant" OR "Neoadjuvant Chemotherapies")) OR (AB=("Neoadjuvant Therapy" OR "Neoadjuvant Therapies" OR "Therapy, Neoadjuvant" OR "Neoadjuvant Treatment" OR "Neoadjuvant Treatments" OR "Treatment, Neoadjuvant" OR "Neoadjuvant Chemotherapy" OR "Chemotherapy, Neoadjuvant" OR "Neoadjuvant Chemotherapies"))) AND( (TS=("Esophageal Neoplasms" OR "Esophageal Neoplasm" OR "Neoplasm, Esophageal" OR "Esophagus Neoplasm" OR "Esophagus Neoplasms" OR "Neoplasm, Esophagus" OR "Neoplasms, Esophagus" OR "Neoplasms, Esophageal" OR "Cancer of Esophagus" OR "Esophageal Cancer" OR "Cancer, Esophageal" OR "Cancers, Esophageal" OR "Esophageal Cancers" OR "Cancer of the Esophagus" OR "Esophagus Cancer" OR "Cancer, Esophagus" OR "Cancers, Esophagus" OR "Esophagus Cancers")) OR (TI=("Esophageal Neoplasms" OR "Esophageal Neoplasm" OR "Neoplasm, Esophageal" OR "Esophagus Neoplasm" OR "Esophagus Neoplasms" OR "Neoplasm, Esophagus" OR "Neoplasms, Esophagus" OR "Neoplasms, Esophageal" OR "Cancer of Esophagus" OR "Esophageal Cancer" OR "Cancer, Esophageal" OR "Cancers, Esophageal" OR "Esophageal Cancers" OR "Cancer of the Esophagus" OR "Esophagus Cancer" OR "Cancer, Esophagus" OR "Cancers, Esophagus" OR "Esophagus Cancers")) OR (AB=("Esophageal Neoplasms" OR "Esophageal Neoplasm" OR "Neoplasm, Esophageal" OR "Esophagus Neoplasm" OR "Esophagus Neoplasms" OR "Neoplasm, Esophagus" OR "Neoplasms, Esophagus" OR "Neoplasms, Esophageal" OR "Cancer of Esophagus" OR "Esophageal Cancer" OR "Cancer, Esophageal" OR "Cancers, Esophageal" OR "Esophageal Cancers" OR "Cancer of the Esophagus" OR "Esophagus Cancer" OR "Cancer, Esophagus" OR "Cancers, Esophagus" OR "Esophagus Cancers"))) AND( (TS=("Immune Checkpoint Inhibitors" OR "Checkpoint Inhibitors, Immune" OR "Immune Checkpoint Blockers" OR "Checkpoint Blockers, Immune" OR "Immune Checkpoint Inhibitor" OR "Checkpoint Inhibitor, Immune")) OR (TI=("Immune Checkpoint Inhibitors" OR "Checkpoint Inhibitors, Immune" OR "Immune Checkpoint Blockers" OR "Checkpoint Blockers, Immune" OR "Immune Checkpoint Inhibitor" OR "Checkpoint Inhibitor, Immune")) OR (AB=("Immune Checkpoint Inhibitors" OR "Checkpoint Inhibitors, Immune" OR "Immune Checkpoint Blockers" OR "Checkpoint Blockers, Immune" OR "Immune Checkpoint Inhibitor" OR "Checkpoint Inhibitor, Immune"))) |
| **EMBASE**  The database was searched on November 5, 2025, n=110.  Search Strategy:  ( 'esophageal cancer'/de OR 'esophageal neoplasm':ti,ab OR 'neoplasm, esophageal':ti,ab OR 'esophagus neoplasm':ti,ab OR 'esophagus neoplasms':ti,ab OR 'neoplasm, esophagus':ti,ab OR 'neoplasms, esophagus':ti,ab OR 'neoplasms, esophageal':ti,ab OR 'cancer of esophagus':ti,ab OR 'esophageal cancer':ti,ab OR 'cancer, esophageal':ti,ab OR 'cancers, esophageal':ti,ab OR 'esophageal cancers':ti,ab OR 'cancer of the esophagus':ti,ab OR 'esophagus cancer':ti,ab OR 'cancer, esophagus':ti,ab OR 'cancers, esophagus':ti,ab OR 'esophagus cancers':ti,ab ) AND ( 'neoadjuvant therapy'/de OR 'neoadjuvant chemotherapy'/de OR 'neoadjuvant therapies':ti,ab OR 'therapy, neoadjuvant':ti,ab OR 'neoadjuvant treatment':ti,ab OR 'neoadjuvant treatments':ti,ab OR 'treatment, neoadjuvant':ti,ab OR 'neoadjuvant chemotherapy':ti,ab OR 'chemotherapy, neoadjuvant':ti,ab OR 'neoadjuvant chemotherapies':ti,ab OR 'neoadjuvant chemotherapy treatment':ti,ab OR 'chemotherapy treatment, neoadjuvant':ti,ab OR 'neoadjuvant chemotherapy treatments':ti,ab OR 'treatment, neoadjuvant chemotherapy':ti,ab ) AND ( 'immune checkpoint inhibitor'/de OR 'checkpoint inhibitors, immune':ti,ab OR 'immune checkpoint blockers':ti,ab OR 'checkpoint blockers, immune':ti,ab OR 'immune checkpoint inhibitor':ti,ab OR 'checkpoint inhibitor, immune':ti,ab )AND('randomized controlled trial'/de OR 'randomized controlled trial':ti,ab OR 'randomised controlled trial':ti,ab OR 'randomized':ti,ab OR 'randomised':ti,ab OR 'randomization':ti,ab OR 'randomisation':ti,ab OR 'placebo':ti,ab OR 'randomly':ti,ab OR 'clinical trial':ti,ab OR 'rct':ti,ab) |
| **Cochrane Library**  The database was searched on November 5, 2025,n=9.  Search Strategy: ("esophageal cancer" OR "esophageal neoplasm" OR "esophagus neoplasm" OR "cancer of esophagus" OR "esophageal cancers" OR "esophagus cancers" OR "neoplasms, esophagus" OR "neoplasms, esophageal" OR "cancer, esophageal" OR "cancer, esophagus") in Title Abstract Keyword AND ("neoadjuvant therapy" OR "neoadjuvant chemotherapy" OR "neoadjuvant treatment" OR "neoadjuvant treatments" OR "chemotherapy, neoadjuvant" OR "neoadjuvant chemotherapy" OR "neoadjuvant chemotherapy treatment") in Title Abstract Keyword AND ("immune checkpoint inhibitor" OR "checkpoint inhibitors, immune" OR "immune checkpoint blockers" OR "checkpoint blockers, immune" OR "immune checkpoint inhibitor" OR "checkpoint inhibitor, immune") in Title Abstract Keyword AND ("randomized controlled trial" OR "randomized controlled trial" OR "randomised controlled trial" OR "rct" OR "randomization" OR "randomised" OR "randomisation" OR "clinical trial" OR "placebo" OR "randomly") in Title Abstract Keyword - (Word variations have been searched) |
| **ScienceDirect**  The database was searched on November 5, 2025, n=698.  Search Strategy:  Title,abstract,keywords(("immune checkpoint inhibitor" OR "PD-1 inhibitor" OR "PD-L1 inhibitor") AND ("Esophageal squamous cell carcinoma" OR ESCC OR "Esophageal cancer" OR "Oesophageal Squamous Cell Carcinoma") AND neoadjuvant therapy AND Randomized |
| **Scoups**  The database was searched on November 5, 2025, n=138.  Search Strategy:  TITLE-ABS-KEY ("immune checkpoint inhibitor" OR "immune checkpoint blockade" OR immunotherapy OR "PD-1 inhibitor" OR "PD-L1 inhibitor") AND TITLE-ABS-KEY("esophageal cancer" OR "oesophageal cancer" OR "esophageal squamous cell carcinoma" OR "oesophageal squamous cell carcinoma" OR ESCC) AND TITLE-ABS-KEY (neoadjuvant OR "neoadjuvant therapy" OR "neoadjuvant chemotherapy" OR preoperative) AND TITLE-ABS-KEY (randomized OR randomised OR randomly OR randomization OR "randomized controlled trial" OR RCT) |

**Note:** The combined text and medical subject heading (MeSH) terms used were: “neoadjuvant chemotherapy with immunothrapy”, “esophageal squamous cell carcinoma”, and “randomized controlled trail”.
