# Supplementary material for: Efficacy and safety of neoadjuvant chemotherapy with immunotherapy versus chemotherapy alone in esophageal squamous cell carcinoma: a meta-analysis based on randomized controlled trials
Source: Front Immunol. 2026 Jul 9;17:1825905. doi: 10.3389/fimmu.2026.1825905 (PMC13391947; doi:10.3389/fimmu.2026.1825905)
Supplement: Supplementary file 6 [file Table2.docx]

| **Outcome** | **Analysis type** | **Factor** | **Subgroup** | **n** | **Effect estimate**  **(95% CI)** | **I²**  **(%)** | **P for subgroup difference** |
| --- | --- | --- | --- | --- | --- | --- | --- |
| MPR | Primary subgroup analysis | Chemotherapy regimen | Paclitaxel-based | 3 | OR 2.76 (1.16, 6.56) | 73 | 0.74 |
| MPR | Primary subgroup analysis | Chemotherapy regimen | Nab-paclitaxel-based | 2 | OR 2.17 (1.13, 4.20) | 11 |  |
| MPR | Primary subgroup analysis | Chemotherapy regimen | Docetaxel-based | 1 | OR 1.38 (0.29, 6.60) | NA |  |
| MPR | Primary subgroup analysis | Chemotherapy regimen | Overall | 6 | OR 2.40 (1.45, 3.98) | 50 | NA |
| MPR | Primary subgroup analysis | ICI agent | Camrelizumab | 3 | OR 3.06 (2.06, 4.53) | 0 | 0.03 |
| MPR | Primary subgroup analysis | ICI agent | Nivolumab | 1 | OR 1.00 (0.39, 2.53) | NA |  |
| MPR | Primary subgroup analysis | ICI agent | Socazolimab | 1 | OR 1.36 (0.46, 4.03) | NA |  |
| MPR | Primary subgroup analysis | ICI agent | Toripalimab | 1 | OR 5.69 (2.24, 14.46) | NA |  |
| MPR | Primary subgroup analysis | ICI agent | Overall | 6 | OR 2.40 (1.45, 3.98) | 50 | NA |
| MPR | Sensitivity analysis | Excluding Jiao 2025 | Paclitaxel-based | 2 | OR 3.84 (2.49, 5.92) | 0 | 0.21 |
| MPR | Sensitivity analysis | Excluding Jiao 2025 | Overall | 5 | OR 2.97 (1.94, 4.53) | 23 | NA |
| pCR | Primary subgroup analysis | Chemotherapy regimen | Paclitaxel-based | 3 | OR 4.04 (2.24, 7.29) | 55 | 0.66 |
| pCR | Primary subgroup analysis | Chemotherapy regimen | Nab-paclitaxel-based | 2 | OR 2.70 (1.32, 5.52) | 0 |  |
| pCR | Primary subgroup analysis | Chemotherapy regimen | Docetaxel-based | 1 | OR 5.74 (0.25, 130.37) | NA |  |
| pCR | Primary subgroup analysis | Chemotherapy regimen | Overall | 6 | OR 3.53 (2.26, 5.53) | 15 | NA |
| pCR | Primary subgroup analysis | ICI agent | Camrelizumab | 3 | OR 4.72 (2.52, 8.86) | 0 | 0.14 |
| pCR | Primary subgroup analysis | ICI agent | Nivolumab | 1 | OR 1.15 (0.32, 4.08) | NA |  |
| pCR | Primary subgroup analysis | ICI agent | Socazolimab | 1 | OR 1.85 (0.62, 5.56) | NA |  |
| pCR | Primary subgroup analysis | ICI agent | Toripalimab | 1 | OR 4.81 (1.57, 14.73) | NA |  |
| pCR | Primary subgroup analysis | ICI agent | Overall | 6 | OR 3.53 (2.26, 5.53) | 15 | NA |
| pCR | Sensitivity analysis | Excluding Jiao 2025 | Paclitaxel-based | 2 | OR 5.38 (2.70, 10.71) | 0 | 0.38 |
| pCR | Sensitivity analysis | Excluding Jiao 2025 | Nab-paclitaxel-based | 2 | OR 2.70 (1.32, 5.52) | 0 |  |
| pCR | Sensitivity analysis | Excluding Jiao 2025 | Docetaxel-based | 1 | OR 5.74 (0.25, 130.37) | NA |  |
| pCR | Sensitivity analysis | Excluding Jiao 2025 | Overall | 5 | OR 4.07 (2.51, 6.60) | 0 | NA |
