# Supplementary material for: Efficacy and safety of neoadjuvant chemotherapy with immunotherapy versus chemotherapy alone in esophageal squamous cell carcinoma: a meta-analysis based on randomized controlled trials
Source: Front Immunol. 2026 Jul 9;17:1825905. doi: 10.3389/fimmu.2026.1825905 (PMC13391947; doi:10.3389/fimmu.2026.1825905)
Supplement: Supplementary file 7 [file Table3.docx]

| Study | Phase | Year | Disease stage | Primary endpoint | Groups | Sample size | Patients | Sex (M/F) | Age (mean, year) | ECOG PS 0 | ECOG PS 1 | ECOG PS 2 | ICIs type | Chemotherapy |
| --- | --- | --- | --- | --- | --- | --- | --- | --- | --- | --- | --- | --- | --- | --- |
| Jiao (29) | II | 2025 | *T1N2M0 or T2–T4aN1–N2M0* | pCR | NIC | 90 | 60 | 50,10 | 62 | 6 | 54 | 0 | Nivolumab | Paclitaxel+Cisplatin |
|  |  |  |  |  | NC |  | 30 | 26,4 | 65 | 1 | 29 | 0 | - |  |
| Li(26) | II | 2023 | *T2 N1–N3M0 or T3–T4aN1–N3M0* | MPR | NIC | 64 | 32 | 51,13 | 62 | 25 | 7 | 0 | Socazolimab | Nab-paclitaxel+Cisplatin |
|  |  |  |  |  | NC |  | 32 |  |  | 27 | 5 | 0 | - |  |
| Qin(17) | III | 2024 | *T1b-3N1-N3M0 or T3N0M0* | pCR | NIC | 391 | 262 | 228,334 | 63 | 106 | 24 | 0 | Camrelizumab | Paclitaxel+Cisplatin |
|  |  |  |  |  | NC |  | 129 | 104,25 | 65 | 104 | 25 | 0 | - |  |
| Wang(28) | II | 2023 | *T2N0-N1M0 or T3~4aN1~2M0* | Tumor response assessed by RECIST 1.1 | NIC | 30 | 15 | 13,2 | 61 | 15 | 0 | 0 | Camrelizumab | Docetaxel+Cisplatin+Fluorouracil |
|  |  |  |  |  | NC |  | 15 | 15,0 | 63 | 15 | 0 | 0 | - |  |
| Zhang(27) | III | 2023 | *cT1-T4N1-N3M0 or cT3-T4N0M0* | pCR and 5-years OS | NIC | 150 | 90 | 128,22 | 65 | 22 | 68 | 0 | Camrelizumab | Nab-paclitaxel+Cisplatin |
|  |  |  |  |  | NC |  | 60 |  |  | 14 | 46 | 0 | - |  |
| Zheng(16) | III | 2024 | *T1N1-N3M0 or T2-3N0-3M0* | EFS | NIC | 252 | 127 | 97,30 | 66 | 98 | 27 | 2 | Toripalimab | Paclitaxel+Cisplatin |
|  |  |  |  |  | NC |  | 125 | 97,28 | 68 | 105 | 20 | 0 | - |  |

Table1

Abbreviations: ECOG PS: Eastern Cooperative Oncology Group Performance Status; M/F: Male/Female; NC: Neoadjuvant chemotherapy; NIC: Neoadjuvant chemotherapy with PD-1/PD-L1 inhibitors; ICIs: Immune checkpoint inhibitors; MPR: Major pathological response;pCR: Pathological complete response;RECIST: Response evaluation criteria in solid tumors ; EFS: Event-free survival; OS: Overall survival.
