# Supplementary material for: Efficacy and safety of neoadjuvant chemotherapy with immunotherapy versus chemotherapy alone in esophageal squamous cell carcinoma: a meta-analysis based on randomized controlled trials
Source: Front Immunol. 2026 Jul 9;17:1825905. doi: 10.3389/fimmu.2026.1825905 (PMC13391947; doi:10.3389/fimmu.2026.1825905)
Supplement: Supplementary file 8 [file Table4.docx]

**Author(s):** yibang Ye

**Question:** Neoadjuvant Chemotherapy with Immunotherapy compared to Neoadjuvant Chemotherapy for Esophageal Squamous Cell Carcinoma

**Setting:**

**Bibliography:**

| **Certainty assessment** | | | | | | | **№ of patients** | | **Effect** | | **Certainty** | **Importance** |
| --- | --- | --- | --- | --- | --- | --- | --- | --- | --- | --- | --- | --- |
| **№ of studies** | **Study design** | **Risk of bias** | **Inconsistency** | **Indirectness** | **Imprecision** | **Other considerations** | **Neoadjuvant Chemotherapy with Immunotherapy** | **Neoadjuvant Chemotherapy** | **Relative (95% CI)** | **Absolute (95% CI)** |  |  |
| **MPR** | | | | | | | | | | | | |
| 6 | randomised trials | not serious | not serious | not serious | not serious | none | 239/558 (42.8%) | 78/351 (22.2%) | **OR 2.40** (1.45 to 3.98) | **185 more per 1,000** (from 71 more to 310 more) | ⨁⨁⨁⨁ High | CRITICAL |
| **pCR** | | | | | | | | | | | | |
| 6 | randomised trials | not serious | not serious | not serious | not serious | none | 124/558 (22.2%) | 28/351 (8.0%) | **OR 3.53** (2.26 to 5.53) | **155 more per 1,000** (from 84 more to 244 more) | ⨁⨁⨁⨁ High | CRITICAL |
| **OS** | | | | | | | | | | | | |
| 2 | randomised trials | not serious | not serious | not serious | serious^a^ | none | -/187 | -/155 | **HR 0.57** (0.32 to 1.01) | **-- per 1,000** (from -- to --) | ⨁⨁⨁◯ Moderate^a^ | CRITICAL |
| **EFS** | | | | | | | | | | | | |
| 2 | randomised trials | not serious | not serious | not serious | serious^a^ | none | -/187 | -/155 | **HR 0.72** (0.49 to 1.06) | **-- per 1,000** (from -- to --) | ⨁⨁⨁◯ Moderate^a^ | CRITICAL |
| **R0 resection** | | | | | | | | | | | | |
| 4 | randomised trials | not serious | not serious | not serious | not serious | none | 408/416 (98.1%) | 240/250 (96.0%) | **OR 2.56** (1.01 to 6.50) | **24 more per 1,000** (from 0 fewer to 34 more) | ⨁⨁⨁⨁ High | CRITICAL |
| **Surgery rate** | | | | | | | | | | | | |
| 4 | randomised trials | not serious | not serious | not serious | not serious | none | 416/481 (86.5%) | 250/316 (79.1%) | **OR 1.57** (1.07 to 2.31) | **65 more per 1,000** (from 11 more to 106 more) | ⨁⨁⨁⨁ High | CRITICAL |
| **Time from last neoadjuvant dose to definitive surgery** | | | | | | | | | | | | |
| 4 | randomised trials | not serious | very serious^b^ | not serious | not serious | none | 416 | 250 | - | MD **4.73 higher** (1.56 higher to 7.9 higher) | ⨁⨁◯◯ Low^b^ | IMPORTANT |
| **The number of lymph nodes resected** | | | | | | | | | | | | |
| 4 | randomised trials | not serious | not serious | not serious | not serious | none | 416 | 250 | - | MD **1.76 higher** (0.74 higher to 2.79 higher) | ⨁⨁⨁⨁ High | IMPORTANT |
| **T Staging** | | | | | | | | | | | | |
| 2 | randomised trials | not serious | not serious | not serious | serious^a^ | none | 77/252 (30.6%) | 59/177 (33.3%) | **OR 0.84** (0.51 to 1.39) | **38 fewer per 1,000** (from 130 fewer to 77 more) | ⨁⨁⨁◯ Moderate^a^ | IMPORTANT |
| **Total AEs** | | | | | | | | | | | | |
| 4 | randomised trials | not serious | serious^c^ | not serious | not serious | none | 444/474 (93.7%) | 295/316 (93.4%) | **RR 1.03** (0.99 to 1.07) | **28 more per 1,000** (from 9 fewer to 65 more) | ⨁⨁⨁◯ Moderate^c^ | CRITICAL |
| **Grade 3 - 4 AEs** | | | | | | | | | | | | |
| 5 | randomised trials | not serious | not serious | not serious | not serious | none | 124/519 (23.9%) | 74/361 (20.5%) | **RR 1.10** (0.87 to 1.40) | **20 more per 1,000** (from 27 fewer to 82 more) | ⨁⨁⨁⨁ High | CRITICAL |
| **iRAEs** | | | | | | | | | | | | |
| 4 | randomised trials | not serious | serious^d^ | not serious | not serious | none | 110/474 (23.2%) | 4/346 (1.2%) | **RR 16.92** (6.55 to 43.75) | **184 more per 1,000** (from 64 more to 494 more) | ⨁⨁⨁◯ Moderate^d^ | CRITICAL |
| **Serious AEs** | | | | | | | | | | | | |
| 4 | randomised trials | not serious | very serious^e^ | not serious | not serious | none | 421/474 (88.8%) | 267/316 (84.5%) | **RR 1.05** (1.00 to 1.10) | **42 more per 1,000** (from 0 fewer to 84 more) | ⨁⨁◯◯ Low^e^ | CRITICAL |
| **AEs leading to discontinuation** | | | | | | | | | | | | |
| 3 | randomised trials | not serious | not serious | not serious | not serious | none | 11/414 (2.7%) | 5/286 (1.7%) | **RR 1.45** (0.48 to 4.44) | **8 more per 1,000** (from 9 fewer to 60 more) | ⨁⨁⨁⨁ High | CRITICAL |

TABLE 2 CI: confidence interval; HR: hazard ratio; MD: mean difference; OR: odds ratio; RR: risk ratio

#### Explanations

a. Downgraded due to imprecision (limited number of studies

b. I² = 95%

c. I² = 77%

d. I² = 76%

e. I² = 92%
