# Supplementary material for: Efficacy and safety of neoadjuvant chemotherapy with immunotherapy versus chemotherapy alone in esophageal squamous cell carcinoma: a meta-analysis based on randomized controlled trials
Source: Front Immunol. 2026 Jul 9;17:1825905. doi: 10.3389/fimmu.2026.1825905 (PMC13391947; doi:10.3389/fimmu.2026.1825905)
Supplement: Supplementary file 9 [file Table5.docx]

| **Adverse Events** | **NIC** | | **NC** | | **Risk ratio [95% CI]** | **P** |
| --- | --- | --- | --- | --- | --- | --- |
|  | **Event/Total** | **%** | **Event/Total** | **%** |  |  |
| Total AEs | 444/474 | 93.67 | 295/316 | 93.35 | 1.03 [0.99, 1.07] | P = 0.19 |
| Grade 3 - 4 AEs | 124/519 | 40.33 | 74/361 | 20.50 | 1.10 [0.87, 1.40] | P = 0.41 |
| iRAEs | 110/474 | 23.21 | 4/346 | 1.16 | 16.92 [6.55, 43.75] | P<0.00001 |
| Serious AEs | 421/474 | 88.82 | 267/316 | 84.49 | 1.05 [1.00, 1.10] | P = 0.06 |
| AEs leading to discontinuation | 11/414 | 2.66 | 5/286 | 1.75 | 1.45 [0.48, 4.44] | P = 0.51 |

TABLE 3
