# Supplementary material for: Efficacy and safety of neoadjuvant chemotherapy with immunotherapy versus chemotherapy alone in esophageal squamous cell carcinoma: a meta-analysis based on randomized controlled trials
Source: Front Immunol. 2026 Jul 9;17:1825905. doi: 10.3389/fimmu.2026.1825905 (PMC13391947; doi:10.3389/fimmu.2026.1825905)
Supplement: Supplementary file 10 [file Table6.docx]

| **Factor** | **Subgroup** | **n** | **Effect estimate**  **(95% CI)** | **I²**  **(%)** | **P for subgroup difference** |
| --- | --- | --- | --- | --- | --- |
| Study phase | Phase II | 2 | MD 7.13 (−7.11, 21.37) | 89 | 0.82 |
| Study phase | Phase III | 2 | MD 5.37 (1.36, 9.39) | 96 |  |
| Treatment duration | 2 cycles | 3 | MD 3.79 (0.61, 6.98) | 96 | 0.03 |
| Treatment duration | 3-4 cycles | 1 | MD 15.25 (5.67, 24.83) | NA |  |
| Overall | All studies | 4 | MD 4.73 (1.56, 7.90) | 95 | NA |

Table S2. Subgroup analyses of time from last neoadjuvant dose to definitive surgery

Abbreviations: CI = Confidence Interval; MD = Mean Difference; NA = Not Applicable.
