# Supplementary material for: Efficacy and safety of neoadjuvant chemotherapy with immunotherapy versus chemotherapy alone in esophageal squamous cell carcinoma: a meta-analysis based on randomized controlled trials
Source: Front Immunol. 2026 Jul 9;17:1825905. doi: 10.3389/fimmu.2026.1825905 (PMC13391947; doi:10.3389/fimmu.2026.1825905)
Supplement: Supplementary file 11 [file Table7.docx]

| Outcome | Factor | Subgroup | n | Effect estimate  (95% CI) | I²  (%) | P for subgroup difference |
| --- | --- | --- | --- | --- | --- | --- |
| iRAEs | Study phase | Phase II | 2 | RR 11.08 (0.22, 556.60) | 85 | 0.94 |
| iRAEs | Study phase | Phase III | 2 | RR 13.46 (0.38, 474.48) | 75 |  |
| iRAEs | Treatment duration | 2 cycles | 3 | RR 21.17 (2.08, 215.48) | 58 | 0.12 |
| iRAEs | Treatment duration | 3-4 cycles | 1 | RR 2.67 (0.78, 9.15) | NA |  |
| iRAEs | Overall | All studies | 4 | RR 16.92 (6.55, 43.75) | 76 | NA |
| Serious AEs | Study phase | Phase II | 2 | RR 1.47 (0.14, 14.93) | 95 | 0.77 |
| Serious AEs | Study phase | Phase III | 2 | RR 1.03 (0.85, 1.25) | 94 |  |
| Serious AEs | Treatment duration | 2 cycles | 3 | RR 1.02 (0.94, 1.10) | 87 | 0.15 |
| Serious AEs | Treatment duration | 3-4 cycles | 1 | RR 2.25 (0.77, 6.57) | NA |  |
| Serious AEs | Overall | All studies | 4 | RR 1.05 (1.00, 1.10) | 92 | NA |
| Total AEs | Study phase | Phase II | 2 | RR 1.00 (0.96, 1.04) | 0 | 0.77 |
| Total AEs | Study phase | Phase III | 2 | RR 1.03 (0.85, 1.25) | 94 |  |
| Total AEs | Treatment duration | 2 cycles | 3 | RR 1.02 (0.94, 1.10) | 87 | 0.74 |
| Total AEs | Treatment duration | 3-4 cycles | 1 | RR 1.00 (0.94, 1.06) | NA |  |
| Total AEs | Overall | All studies | 4 | RR 1.03 (0.99, 1.07) | 77 | NA |

Abbreviations: AE = Adverse Event; CI = Confidence Interval; iRAEs = Immune-Related Adverse Events; MD = Mean Difference; NA = Not Applicable; RR = Risk Ratio.

Table S3. Subgroup analyses of adverse event outcomes
