# Supplementary material for: Efficacy and safety of neoadjuvant chemotherapy with immunotherapy versus chemotherapy alone in esophageal squamous cell carcinoma: a meta-analysis based on randomized controlled trials
Source: Front Immunol. 2026 Jul 9;17:1825905. doi: 10.3389/fimmu.2026.1825905 (PMC13391947; doi:10.3389/fimmu.2026.1825905)
Supplement: Supplementary file 12 [file Table8.docx]

| **Adverse Events** | **NIC** | | **NC** | | **Risk ratio [95% CI]** | **P** |
| --- | --- | --- | --- | --- | --- | --- |
|  | **Event/Total** | **%** | **Event/Total** | **%** |  |  |
| **General Symptoms and Conditions** |  |  |  |  |  |  |
|  |  |  |  |  |  |  |
| Fatigue | 157/429 | 36.60% | 135/301 | 44.85% | 1.12 (1.00, 1.25) | 0.04 |
| Pain | 83/382 | 21.73% | 73/254 | 28.74% | 1.13 (0.91, 1.40) | 0.26 |
| Fever | 32/414 | 7.73% | 21/286 | 7.34% | 1.48 0.88, 2.50) | 0.14 |
| **Hematological Abnormalities** |  |  |  |  |  |  |
|  |  |  |  |  |  |  |
| Anemia | 147/369 | 39.84% | 74/202 | 36.63% | 1.24 (1.02, 1.50) | 0.03 |
| Leukopenia | 153/369 | 41.46% | 65/202 | 32.18% | 1.36 (1.09, 1.70) | 0.006 |
| Neutropenia | 146/369 | 39.57% | 66/202 | 32.67% | 1.29 (1.03, 1.61) | 0.02 |
| Lymphopenia | 42/322 | 13.04% | 15/155 | 9.68% | 1.35 (0.07, 2.36) | 0.29 |
| Thrombocytopenia | 69/369 | 18.70% | 40/202 | 19.80% | 1.34 (1.17, 1.46) | 0.03 |
| **Gastrointestinal Symptoms** |  |  |  |  |  |  |
|  |  |  |  |  |  |  |
| Anorexia | 161/474 | 33.95% | 135/316 | 42.72% | 1.16 (1.04, 1.29) | 0.006 |
| Nausea | 189/489 | 38.66% | 157/331 | 47.43% | 1.01 (0.88, 1.15) | 0.90 |
| Vomiting | 78/429 | 18.18% | 65/301 | 21.60% | 0.99 (0.74, 1.08) | 0.94 |
| Diarrhea | 82/429 | 19.11% | 71/301 | 23.59% | 1.10 (0.86, 1.41) | 0.45 |
| Constipation | 60/397 | 15.11% | 64/269 | 23.79% | 0.97 (0.75, 1.26) | 0.82 |
| Abdominal Distention | 31/382 | 8.12% | 26/254 | 10.24% | 1.20 (0.75, 1.92) | 0.45 |
| Dry Mouth | 32/382 | 8.38% | 27/254 | 10.63% | 1.24 (0.79, 1.94) | 0.35 |
| **Surgical Complications** |  |  |  |  |  |  |
|  |  |  |  |  |  |  |
| Perioperative Mortality | 1/89 | 1.12% | 2/59 | 3.39% | 0.41 (0.05, 3.26) | 0.40 |
| Anastomotic Leak | 12/346 | 3.47% | 7/162 | 4.32% | 0.78 (0.33, 1.85) | 0.57 |
| Recurrent Laryngeal | 22/262 | 8.84% | 9/103 | 8.74% | 0.96 (0.46, 2.02) | 0.92 |
| **Pulmonary Complications** |  |  |  |  |  |  |
|  |  |  |  |  |  |  |
| Pneumonia | 38/346 | 10.98% | 17/162 | 10.49% | 0.94 (0.55, 1.61) | 0.83 |
| Pleural Effusion | 20/317 | 6.31% | 10/133 | 7.52% | 0.86 (0.41, 1.79) | 0.69 |
| Respiratory Failure | 2/317 | 0.63% | 1/133 | 0.75% | 0.79 (0.11, 5.60) | 0.81 |
| Atelectasis | 3/17 | 0.95% | 1/133 | 0.75% | 1.0 (0.18, 5.17) | 0.90 |
| Pneumothorax | 1/291 | 0.34% | 1/132 | 0.75% | 0.61 (0.07, 5.17) | 0.65 |
| Intrathoracic Abscess | 2/317 | 0.63% | 1/133 | 0.75% | 0.75 (0.11, 5.60) | 0.81 |
| **Infectious and Severe Complications** |  |  |  |  |  |  |
|  |  |  |  |  |  |  |
| Septic Shock | 4/317 | 1.26% | 0/133 | 0.00% | 2.4 (0.26, 19.41) | 0.46 |
| **Dermatological and Neurological Complications** |  |  |  |  |  |  |
|  |  |  |  |  |  |  |
| Rash | 49/474 | 10.34% | 13/316 | 4.12% | 2.44 (1.39, 4.25) | 0.002 |
| Peripheral Neuropathy | 10/294 | 3.40% | 7/157 | 4.46% | 0.80 (0.34, 1.91) | 0.62 |
| Alopecia | 183/474 | 38.61% | 149/316 | 47.15% | 1.06 (0.93, 1.20) | 0.37 |
| **Endocrine and Cardiovascular Complications** |  |  |  |  |  |  |
|  |  |  |  |  |  |  |
| Hypokalemia | 50/469 | 10.67% | 26/316 | 8.23% | 1.54 (0.98, 2.43) | 0.06 |
| Hyponatremia | 97/469 | 20.68% | 46/316 | 14.56% | 1.87 (1.37, 2.54) | <0.0001 |
| Elevated Serum Creatinine | 37/474 | 7.81% | 19/316 | 6.01% | 1.15 (0.67, 1.95) | 0.62 |
| Thyroid Dysfunction | 53/382 | 13.87% | 0/254 | 0.00% | 42.77 (6.56, 278.67) | <0.0001 |
| Cardiac Dysrhythmia | 9/262 | 3.40% | 3/103 | 2.91% | 1.18 (0.16, 1.05) | 0.80 |

TABLE S4
